# Comparison of Intimate Partner Violence and Correlates at Urgent Care Clinics and an Emergency Department in a Rural Population

**DOI:** 10.3390/ijerph20054554

**Published:** 2023-03-03

**Authors:** Danielle M. Davidov, Kelly K. Gurka, D. Leann Long, Carmen N. Burrell

**Affiliations:** 1 Department of Social and Behavioral Sciences, West Virginia University, Morgantown, WV 26506, USA; 2 Department of Emergency Medicine, West Virginia University, Morgantown, WV 26506, USA; 3 Department of Epidemiology, University of Florida, Gainesville, FL 32611, USA; 4 Department of Biostatistics, University of Alabama at Birmingham, Birmingham, AL 35294, USA; 5 Department of Family Medicine, West Virginia University, Morgantown, WV 26506, USA

**Keywords:** intimate partner violence, urgent care, emergency department, rural, Appalachia, screening, medical record, composite abuse scale

## Abstract

This paper describes the prevalence of and factors associated with intimate partner violence (IPV) in the urgent care setting and an academic emergency department in Appalachia. A questionnaire assessing social support, mental and physical health status, substance use, and intimate partner violence was administered to 236 women seeking care in an academic emergency department or two affiliated urgent care clinics. Data collected were compared to IPV screening data from medical records. Separate logistic regression models were fit to estimate the association between sociodemographic and health-related factors and lifetime physical and sexual intimate partner violence, adjusted for the clinical setting. Of the 236 participating women, 63 were seen in the emergency department and 173 were seen in an urgent care clinic. Emergency department patients were significantly more likely to report lifetime threatened physical, physical, or sexual abuse. Based on medical records, over 20% of participants had not been screened for IPV by clinical staff during their healthcare visit. Of those that were screened, none disclosed IPV, despite a substantial proportion reporting IPV on the survey. Although survey reports of IPV were lower in the urgent care clinics, this remains an important location to introduce screenings and resources.

## 1. Introduction

Each year in the United States (US), over 6.9 million women experience rape, physical assaults, or stalking by an intimate partner [1,2]. Lifetime prevalence estimates of intimate partner violence (IPV) fall between 28% and 36%, and approximately 6% of women experience IPV annually [1,2]. A large body of literature has demonstrated higher rates of IPV among women seeking healthcare [3,4], especially those seen in emergency department (ED) settings [3,4,5,6,7,8,9]. Lifetime prevalence rates as high as 50% and past-year prevalence between 12–36% have been reported in ED-based cross-sectional studies [10]. Research has also shown that a substantial percentage of ED visits made by women are often related, either directly or indirectly, to IPV [11,12,13,14].

Associations between IPV and poor health are well-established. Physical health impacts for women who have experienced IPV include, but are not limited to, severe injuries, stress and pain, digestive problems, eating disorders, neurological damage, and reproductive health problems [3,15,16,17]. Women who have been physically or sexually abused by an intimate partner are more likely to engage in high-risk sexual behaviors [15,16] and are at greater risk for sexually transmitted infections, unintended pregnancy, and induced abortions [15,16,18,19,20,21] Psychological consequences include depression, anxiety, post-traumatic stress disorder (PTSD), insomnia, social dysfunction, and substance abuse [17,22,23,24]. IPV-related injuries can range from minor cuts and bruises to more severe injuries, such as gunshot or stab wounds, that require medical treatment for the victim [25].

There is considerable evidence that racial and ethnic minority women and those living in inner-city, urban areas experience significant disparities related to IPV prevalence and associated health consequences, such as depression and substance abuse [26,27,28,29,30,31,32,33]. Less research has focused on IPV among women living in rural settings, despite the fact that they experience risk factors similar to their urban, minority counterparts. Some rural populations report low levels of income and educational attainment, as well as high rates of unemployment and negative health status—which are strong predictors of IPV—even after controlling for race/ethnicity [34,35]. A growing body of literature has demonstrated that IPV occurs in rural areas as often as in non-rural areas [30,35]. However, social and geographic isolation, fewer social and medical support systems, and increased travel times to receive services may pose challenges to appropriately addressing IPV in rural settings [35,36,37]. In fact, Choo and colleagues found that compared with urban EDs, rural EDs had significantly fewer resources in place to address IPV [38]. Furthermore, disparities in access to health and supportive care services may be especially pronounced in medically underserved or geographically isolated communities, such as those within the highly rural Appalachian region.

As a response to healthcare shortages and overcrowding in EDs, urgent care (UC) centers have emerged across rural, urban, and suburban areas to increase the provision of immediate care and basic procedures for acute illnesses and minor injuries. While the usual patient base for UC centers consists of privately insured, healthy, young adults, some UC centers employ a fee-for-service model that provides services to uninsured or underinsured patients. Although UC centers are not intended to serve as a substitute for primary care, in areas with physician shortages, patients without an established primary care provider may utilize UC centers routinely for their healthcare needs. For some women exposed to IPV, UC centers may be their only contact with the health system. Furthermore, complaints commonly addressed in UC centers—injuries such as sprains, closed fractures, minor pain and discomfort, and sexually transmitted infections—are associated with IPV [11,39,40].

When compared to the vast amount of research dedicated to the study of IPV in ED and primary care settings, relatively little is known about the prevalence and correlates of IPV in patients presenting to the UC setting. Furthermore, to the best of our knowledge, no published research exists that examines IPV in UC settings that support rural populations. To best support patients living in rural areas who are exposed to IPV and seek emergent or urgent healthcare, a critical first step is to identify and describe the socioeconomic and health status of this group. Thus, the purpose of the current study was two-fold as follows: (1) to examine lifetime and past-year IPV and associations with health outcomes, physical and mental health status, and substance use in two urgent care clinics in a rural Appalachian state; and (2) to compare data from the UC patients to a sample of ED patients seen in an academically affiliated ED.

## 2. Materials and Methods

### 2.1. Study Design

This was a cross-sectional study consisting of a self-administered survey focused on IPV victimization, physical and mental health status, and substance abuse obtained from a convenience sample of female patients. All study procedures were approved by and conducted in accordance with West Virginia University’s Institutional Review Board.

### 2.2. Study Setting and Population

Data for this study were collected in three clinical sites that are part of the West Virginia University (WVU) Medicine healthcare system, namely the emergency department (ED) at J. W. Ruby Memorial Hospital and two academically affiliated urgent care (UC) centers. The WVU Department of Emergency Medicine provides extensive, comprehensive emergency care to sick and injured patients from all parts of West Virginia, southwestern Pennsylvania, Ohio, and Maryland. Over 48,000 patients are treated annually in a department that places emphasis on support of the rural population of the area. At the time of the study, the clinical department consisted of 531 beds, a Level I trauma center, and a tertiary referral hospital. Ruby Memorial Hospital also serves as the principal clinical education and research site for the WVU School of Medicine. As an extension of their emergency services, the Department of Emergency Medicine at WVU operates two UC centers near the hospital. Each of these UC centers treat approximately 25,000 patients annually. Immediate acute care for illnesses and minor injuries is provided during normal business hours, evenings, and weekends to patients aged three months and older.

These three clinical sites serve the highly rural population of West Virginia, handling a large caseload of chronic disease, substance abuse, and injury. Although racially and ethnically homogenous (96% white), the Appalachian state faces many socioeconomic and health disparity conditions akin to those experienced by disadvantaged minority groups and those living in inner-city, urban areas. Compared to national estimates, West Virginians report significantly lower levels of income and educational attainment, higher rates of chronic disease morbidity and mortality, and increased health risk behaviors, such as smoking and drug overdose deaths [41]. WV also leads the nation in poor health status, with residents reporting significantly more poor physical health days (4.9 days) and mental health days (4.5 days) per month than the national average (2.7 and 3.5 days, respectively). Additionally, from 1990 to 2014, violent crime increased by 44% in WV (from 219 to 316 offenses per 100,000 population) and one-third of all homicides in the state are related to domestic violence [42].

### 2.3. Study Procedures

Female patients presenting to one of the three study sites who were 18 years of age or older, not critically ill, and had the capacity to consent were eligible to participate. Due to a lack of a private, dedicated research space in each of the clinical settings, patients were approached in their private rooms. To maximize confidentiality and safety, patients accompanied by friends, family, or significant others were not approached to participate. Data were collected by trained research assistants, most of whom were medical and public health students enrolled in a clinical research methods course. They worked varying shifts in the ED and UC centers, typically Monday through Friday, 9 a.m.–7 p.m., but hours varied by time of year and semester. Based on research assistant availability, some of the data collection occurred on weekends and late evening hours. All research assistants underwent rigorous training in the protection of human subjects (i.e., HIPAA, OSHA), as well as on informed consent and safety procedures for collecting sensitive data. Our team employed various strategies (described below) to ensure that participants felt safe and comfortable being approached for participation by members of the research team, who were a mix of men and women, typically between the ages of 18–24 years.

Before implementation, 13 students provided feedback on the survey to ensure clarity and completeness of the consent process and survey questions. The survey was pilot tested at the clinical sites for two weeks—patients provided reactions to the survey and research assistants provided feedback to the research team during a debriefing session. Based on this information, modifications were made to the original survey, and data collection began in April 2012 and ended in December 2014. To enroll patients in the study, research assistants first communicated with members of the clinical staff to determine eligibility and ensure that the patient was alone. Research assistants consulted staff members to determine if patients might be agreeable to being approached for participation. For example, the research team agreed that patients who were in apparent distress, seriously ill, or who were sleeping should not be approached. Furthermore, in order to ensure that potential participants felt safe being approached and understood that their involvement in the project was voluntary, a member of the clinical staff spoke with the patient prior to recruitment to seek permission for a student researcher to speak with them about a survey opportunity. Only after the patient gave permission did the research team approach them for potential participation. Although participation could have occurred at any point during their medical visit, the research team spoke with patients after intake procedures and while they were waiting for test results most of the time.

Once clinical staff members confirmed that a patient agreed to hear about the research, eligible patients were approached by research assistants and asked to confirm they would be alone for the duration of their visit. Then, they asked if they would be interested in learning more about an opportunity to participate in a study about “health and relationship experiences”, which included questions about violence in intimate relationships, physical and mental health, and drug use. In line with standard informed consent procedures, research assistants shared that the project was being conducted as part of a clinical research class and had no impact on their clinical care and that their medical team would not see their answers or know whether or not they chose to participate unless they shared that information. They were informed that participation was voluntary and were given an opportunity to see the survey before deciding whether or not to participate. They were instructed that they could skip any question they did not want to answer and could stop participating at any time.

Interested patients were able to view and discuss the consent form with the research assistant before agreeing to participate. As part of the consent process, research assistants preemptively discussed that due to the sensitive nature of the survey content, resources were available if they felt uncomfortable, upset, or experienced an adverse or emotional reaction. To protect patient safety, we did not provide a copy of the resources or the signed consent form unless the patient requested them; the research assistants always asked patients if they felt it was safe to take the resource lists/consent form home with them if they were requested. Participants were also informed that access to medical providers and social workers was available if needed. Patients who provided written informed consent completed a paper-and-pencil survey and were asked to place it in a sealed envelope to protect their confidentiality. After 20 min, the research assistant checked back with the patient to collect the completed survey.

### 2.4. Measures

The survey instrument captured information on sociodemographic characteristics; social support, mental and physical health status; tobacco, alcohol, and drug use; and lifetime and past-year or past-partner violence victimization. Standardized measures with known psychometric properties that have been tested with clinical samples were used almost exclusively in the survey. Much of the survey instrument was developed using questions from the Behavioral Risk Factor Surveillance System (BRFSS) survey, which has demonstrated moderate-to-high reliability and validity in comparison studies [43].

#### 2.4.1. Sociodemographic Characteristics

Sociodemographic variables were adopted from the BRFSS questions for age, race/ethnicity, marital status, military history, educational and employment status, and annual household income. In addition to the question about marital status, an item was added about relationship status to assess cohabitation among unmarried participants. A question from the National Health Interview Survey that asked participants where they usually go when they are sick or need advice about their health was also included.

#### 2.4.2. Social Support and Mental and Physical and Health Status

Social support was measured using items from a previous study that examined associations between IPV, substance abuse, and depression in an urban sample of African American women [32]. Questions asked if participants had daily contact with other people, if there was someone in their life they could talk to about any problem, if they had someone to stay with in case of emergency, and if they usually have enough money to meet their needs. Response choices for each of these four items were yes and no. Self-rated health was assessed by asking respondents to rate their general health as excellent, very good, good, fair, or poor. Disability status was assessed using the BRFSS question, “Are you limited in any way in any activities because of physical, mental, or emotional problems” with response choices of yes or no. Mental health status was measured with two separate questions asking if the participant had ever been told by a doctor or other healthcare provide that they had an anxiety or depressive disorder, with response choices of yes or no.

#### 2.4.3. Substance Use

Substance use was assessed using three well-validated and reliable measures that have shown high sensitivity and specificity in general population and clinical samples. Tobacco use was measured with two questions from the modified version of the Heavy Smoking Index (HSI-M), which asks “Do you smoke cigarettes or use any other form of tobacco?” and “Do you smoke or use tobacco every day?” [44]. Alcohol abuse was assessed using the Alcohol Use Disorders Identification Test-Consumption (AUDIT-C), which is a three-item screening tool that assesses the presence of alcohol use disorders or risky drinking behaviors. It is scored on a scale of 0–12 with a score of ≥3 indicating a positive screen for females. The 10-item Drug Abuse Screening Tool (DAST-10) was used to assess drug use in the past 12 months. This measure is scored from 0–10 and a score of ≥3 indicates a positive screen for drug abuse.

#### 2.4.4. Intimate Partner Violence

Survey Data. Lifetime and past-year physical and sexual intimate partner violence were measured with the following questions from the BRFSS IPV module: (1) “Has an intimate partner ever hit, slapped, pushed, kicked, or physically hurt you in any way?” (lifetime physical IPV); (2) “Has an intimate partner ever threatened you with physical violence? This includes threatening to hit, slap, push, kick, or physically hurt you in any way?” (lifetime threatened physical IPV); (3) “Have you ever experienced unwanted sex by a current or former intimate partner?” (lifetime sexual IPV); (4) “In the past 12 months, have you experienced any physical violence or had unwanted sex with an intimate partner?” (past-year physical or sexual IPV); and (5) “In the past 12 months, have you had any physical injuries, such as bruises, cuts, scrapes, black eyes, vaginal or anal tears, or broken bones, as a result of this physical violence or unwanted sex?” (past-year IPV-related injuries). The response categories for each of these questions was yes, no, or I prefer not to answer.

We received permission to use the Composite Abuse Scale (CAS) to assess past-year/past-partner IPV [45]. Well-validated and widely used in primary care and ED settings, this 30-item measure provides information on the type, frequency, and severity of abuse experienced in the past 12 months. For each of the 30 items, participants select how often the behavior occurred, on a scale from never, only once, several times, once a month, once a week, to daily. The items are grouped into four separate subscales as follows: emotional abuse (e.g., told me that I was not good enough, blamed me for causing their violence behavior); harassment (e.g., followed me, hung around outside my house); physical abuse (i.e., pushed, grabbed or shoved me, beat me up); and severe combined abuse (e.g., kept me from medical care, raped me, or used a knife, gun, or other weapon). The CAS has demonstrated good internal reliability and high face, content, criterion, and construct validity [46,47,48]. Respondents were asked to complete the CAS questions based on whether or not the actions happened to them over the past 12 months and, if they were not with a partner in the last 12 months, they were asked to answer for the last partner they had.

Medical Record Review. In addition to survey data on IPV, we received permission from each participant to review their medical records to determine whether IPV screening questions were asked during their clinical visit and to examine their responses.

### 2.5. Statistical Analysis

To compare study participant characteristics across the different care settings (data from both UC clinics compared with the ED setting), Fisher’s exact tests and Wilcoxon rank-sum tests were used for the nominal categorical variables and continuous variables, respectively. The underlying assumption of the Wilcoxon rank-sum test is the alternative of location shift in the continuous variable, which focuses on empirical distribution rather than the mean specifically. Fisher’s exact test assumes the table margins are fixed. For the ordinal characteristics, such as education level, income level, and health status, Mantel–Haenszel chi-squared tests were used to investigate potential differences in trend across the care settings [49]. To estimate the association of each characteristic with lifetime physical or sexual IPV (hereafter referred to as lifetime IPV) in the study population, separate logistic regression models were fitted for each characteristic accounting for the clinical setting, and Wald chi-squared tests were used to assess statistical significance. Logistic regression assumptions included binomial distribution of lifetime IPV for each combination of covariates (care setting and each patient characteristic). In an exploratory analysis, the statistical interactions between setting (UC vs. ED) and each characteristic were used to examine the potential effect measure modification of the care setting on the odds of lifetime IPV. All statistical analyses were performed in SAS 9.3 (SAS Institute, Inc., Cary, NC, USA).

## 3. Results

Overall, 274 women were approached to participate at the three clinical care sites—the ED and two UC clinics. Among those approached, 89 were eligible to participate in the ED, with an additional 185 eligible to participate at the UC centers. Of those, 63 (70.8%) women in the ED and 173 (93.5%) women in the UC centers who were invited to participate completed the survey (total = 236) for an overall response rate of 86.1%.

### 3.1. Sociodemographics

Patients who completed the survey were generally young (median age = 29 years, IQR: 21, 44), but women participating in the ED were significantly older (*p* = 0.02) than those participating in the UC clinics (Table 1). Women participating in the study were homogenous with regard to race and ethnicity, with a majority (94%) of women being non-Hispanic white. Women presenting to UC centers had significantly higher levels of education (*p* < 0.01), were more likely to be employed or a student (*p* < 0.01), and reported higher income levels (*p* < 0.01) than women presenting to the ED.

### 3.2. Social Support and Health Status

Women also differed regarding social support and health status. Though high among both groups, women presenting to the UC centers were significantly more likely to report having good social support (Table 2). Women utilizing the UC centers were also more likely to report higher ratings of self-perceived health status compared to those utilizing the ED (*p* < 0.01). Nearly half of the women surveyed in the ED reported limitations in their daily activities due to health compared to only about a fifth of women surveyed in the UC centers (*p* < 0.01). Women in the ED were also significantly more likely to report both anxiety and depression (*p* < 0.01 and *p* = 0.01, respectively). Though women surveyed in the ED reported more tobacco use (27% versus 19%) and drug abuse (6% versus 4%), women surveyed in the UC centers were significantly more likely to be classified as abusing alcohol based on the AUDIT-C than their ED counterparts (*p* = 0.01).

### 3.3. Intimate Partner Violence

Using the Composite Abuse scale (CAS), women reporting to both care settings were most likely to report emotional abuse in the past year or with their last partner, followed by physical abuse (Table 3). Women surveyed in the ED were significantly more likely to endorse lifetime threatened physical, physical, and sexual abuse compared to their UC counterparts. In addition, women in the ED reported about twice as much past-year physical or sexual abuse and injuries resulting from these forms of abuse compared to women in urgent care (6.5% versus 3.5% and 4.8% versus 1.7%, respectively).

The medical record review revealed that patients were asked seven different types of IPV screening questions during their medical visit (Table 4). The most frequently asked question was “Are you being hurt, hit, or frightened by anyone at your home or in your life?”, which was used to screen 61 patients. Furthermore, 50 patients (21%) were not asked any screening questions. No positive screens for IPV were noted among the patients who were asked the IPV screening questions.

Table 5 displays the odds ratios of lifetime IPV (physical and/or sexual) for sociodemographic, social support, and health status variables, while adjusting for the care setting. Respondents reporting limitations to their daily activities due to health had 2.86 times the odds of experiencing lifetime IPV. Those with anxiety and depression diagnoses had 3.01 and 3.99 times the odds, respectively, of reporting lifetime physical or sexual IPV compared to those respondents without these diagnoses. Those reporting tobacco (OR = 4.08) and drug use (OR = 4.55) were also more likely than those not reporting substance use to have significantly higher odds of lifetime IPV. There were no significant differences in associations between patient characteristics and lifetime IPV between the two care settings (Table A1—Appendix A).

## 4. Discussion

To the best of our knowledge, this is the first study to examine IPV prevalence and correlates in an academically affiliated ED and make comparisons with associated urgent care clinics that support rural populations. As UC centers continue to expand throughout the US and sustain high patient volumes, it is critical to implement best practices for addressing IPV in these settings. This study adds information about the health status and sociodemographic profile of this group to the extant literature. This is especially true for rural or underserved settings where resources may be scarce. Most clinical studies of rural IPV focus on a specific population, such as patients who are pregnant or report substance use disorders [50,51].

The sociodemographic characteristics of patients in our sample match previous epidemiological reports demonstrating high rates of poverty, lower levels of educational attainment, and poorer health status of rural residents compared with their urban counterparts [52,53,54]. Women participating in the study were racially and ethnically homogenous, as the majority (94%) were non-Hispanic white, which is reflective of the larger geographic area where the study was conducted. Differences in sociodemographic variables were found across care settings. Patients seeking care in the ED were significantly older and had lower levels of education, income, and social support compared to those in the UC clinics. This is expected, as UC clinics generally evaluate a younger population who may not be established with a primary care physician for chronic illness. The medical literacy of this population may be higher as they seek care in a quick, convenient, and accessible location for lower acuity health concerns, and the clinics are often located in regions that serve patients with higher income levels. Additionally, urgent care clinics typically treat more patients with private health insurance; only a small proportion use Medicaid as their payor. Still, across both care settings, a significant proportion of participants reported limitations to daily living, as well as adverse mental health and substance abuse outcomes.

In the ED, almost half of the respondents reported significant limitations in their daily activities due to their health. Patients in the ED were also more likely to endorse anxiety or depression diagnoses and meet the criteria for tobacco and drug abuse. Close to one-third of the participants seeking care in the UC clinics reported anxiety or depression diagnoses. They were also more likely than those in the ED to meet the criteria for alcohol abuse. This is likely explained by UC clinics serving high numbers of college students, given that this study took place in a college town with students reporting high rates of alcohol use [55]. Furthermore, prior research has demonstrated that higher levels of socioeconomic status may correlate with higher levels of alcohol use [56]. In line with previous studies demonstrating that women seeking healthcare report high rates of IPV [6,57,58], a substantial proportion of participants in this study experienced all types of abuse. As expected, a significantly higher percentage of women seeking care in the ED reported IPV compared with those in UC clinics. Kramer and colleagues examined IPV prevalence among women seeking emergent and primary care in urban, suburban, and rural settings and found that women presenting to EDs reported the highest rates of physical abuse versus those in primary care clinics [6]. Furthermore, multiple studies have reported strong relationships between IPV and adverse health outcomes, such as substance abuse and mental health disorders, in clinical populations [32,50,51,57,59].

In our study, while adjusting for care setting, those patients reporting limitations in their daily activities due to health, depression and anxiety diagnoses, and tobacco and drug use had significantly higher odds of experiencing lifetime physical or sexual IPV. A study by Hankin and colleagues found that African-American women who screened positive for IPV in urban EDs were more likely to endorse alcohol, tobacco, and drug abuse, as well as report social isolation and depression [32]. Caetano, Cunradi, Alter, and Mair found women’s likelihood of IPV and greater IPV severity occurs with increasing numbers of risk factors (e.g., depression, drug use, at-risk drinking) [60]. In particular, women with four to seven risk factors had 20 times the odds of experiencing an IPV event, emphasizing the importance of multi-risk assessment.

Few studies have delineated relationships between IPV and adverse health outcomes in rural populations. Even fewer have focused on rural Appalachian women seeking clinical care, despite being a vulnerable and high-needs group [51]. While not a clinical sample, Nemeth found that IPV among women in Appalachian Ohio was significantly associated with current smoking [59]. Using the Conflict Tactics Scale-2, Bailey and Daughtery reported rates of IPV over 80% among 104 rural Appalachian pregnant women receiving prenatal care, as well as strong associations with smoking, alcohol, and drug use [50]. Additionally, a pilot study by Shannon and others found rates of IPV among pregnant women receiving treatment for substance use dependence or opioid dependence to be significantly higher than the national average [51]. Our findings add to the gap in the literature by providing new information on past-year/past-partner and lifetime IPV, as well as associations between abuse and sociodemographic and adverse health outcomes in a rural Appalachian clinical sample.

West Virginia and other rural areas are experiencing a healthcare shortage. The urgent care model is often used within primary care clinics in rural areas to increase walk-in availability of healthcare providers. Women experiencing IPV without an established primary care provider may frequent these clinics for IPV and non-IPV-related health concerns. Because they are an extension of emergency care, preventive services and ancillary resources, such as social work and counseling, may not be readily available. It is critical for these care settings to be considered in research and policy surrounding IPV assessment and that they are equipped with appropriate protocols and procedures for addressing IPV, including established connections with community-based supportive services and trauma-informed care for IPV victims and survivors.

Importantly, the medical record review revealed that no patients disclosed IPV during their visit; however, participant survey data demonstrated that almost one-third of patients reported lifetime physical IPV and 4% reported physical or sexual abuse in the past year. Further, 21% of patients were not asked any screening questions. Multiple factors might explain this discrepancy. Although it is unlikely, one explanation is that none of the patients were currently experiencing IPV and answered the survey questions based only on past relationships where IPV was present. However, it is more likely that multiple barriers to IPV disclosure that exist in clinical settings precluded a positive IPV screen. Research has uncovered various provider (e.g., body language, time restrictions, lack of eye contact) and patient (e.g., fear of partner retaliation, language barriers, stigma) barriers to screening that may result in reluctance to discuss IPV and disclose to healthcare providers [61,62,63]. Research carried out by Rhodes and Troutman found similar discrepancies in patient reports of abuse between self-administered computer assessment versus medical record documentation [7,64,65]. Taken with our findings, it is likely that self-report data (especially electronic or written versus verbal) may me more reliable than medical record data, as information documented in medical charts has been shown to underestimate and underreport clinical identification and discussion of IPV [65]. In addition, it may often be nursing or other clinical staff who ask IPV questions and not the care provider.

### Limitations

This study is not without limitations. First, our sample was homogenous regarding race/ethnicity, which precludes us from examining important differences in IPV and health outcomes among different groups, such as women of color, who may experience multiple vulnerabilities that can impact IPV exposure and health. Further, data were collected from three clinical settings in one county in a rural Appalachian state. This county has more healthcare and social service resources than other medically underserved areas in the region. Thus, these data may not be representative of the entire state of West Virginia, rural areas, or Appalachia as a whole. Data were collected via paper-and-pencil surveys versus using an electronic method (e.g., iPad or tablet), which may result in social desirability bias and validity of responses to sensitive questions; however, studies have shown similar rates of IPV disclosure across different screening methods [65]. Additionally, since these data were collected, revisions have been made to the CAS to improve the measure’s items and response options to better capture the complexity of IPV on a continuum. The CAS Revised Short Form [CASR-SF; [66]] is an enhanced valid and reliable measure that draws on the strengths of the original CAS but more thoroughly captures the full spectrum of IPV, while minimizing burden to participants. Although studies are ongoing to further validate the CASR-SF in different contexts and settings, in the future, researchers may wish to use this revised instrument to capture the continuum of women’s experiences of IPV more adequately. This was a convenience sample of patients seeking emergency and urgent care and data collection occurred primarily during daytime hours, with a limited number of patients approached during nighttime or weekend hours. Finally, we restricted study eligibility criteria to those patients who were alone. Patients that seek medical care alone may be different in terms of our outcomes of interest compared with those accompanied by family or friends. In future studies aimed at identifying IPV and associated adverse health outcomes, private spaces should be used for data collection and procedures protecting patient confidentiality should be followed.

## 5. Conclusions

Women enrolled in this study seeking care in the ED were more likely than those at UC clinics to report lifetime physical or sexual IPV, tobacco use, drug abuse, anxiety, and depression. One-third of patients in the UC clinics reported anxiety or depression diagnoses and almost one-quarter reported lifetime physical IPV. These patients were also more likely than those in the ED to meet the criteria for alcohol abuse. While adjusting for the type of care setting, there were strong associations between lifetime IPV prevalence and adverse physical health, mental health, and substance use outcomes. Multi-factorial screening and assessment procedures that maximize patient privacy and confidentiality are critical for addressing IPV rural or underserved areas where urgent care settings may be abused patients’ only routine contact with the healthcare system.

## Figures and Tables

**Table 1:** Sociodemographic characteristics of patients by care setting.

	Total	Emergency Department		Urgent Care		*p*-Value
	*n* (%)	*n*	%	*n*	%	
Age, median (IQR) *	29 (21, 44)	36 (23, 49)		26 (21, 42)		0.0187
Race/Ethnicity						0.7491
Non-Hispanic White	220 (94)	58	92.1	162	93.6	
Hispanic	8 (3)	3	4.8	5	2.9	
Non-Hispanic Other	8 (3)	2	3.1	6	3.5	
Marital Status						0.3273
Never Married, Not Cohabitating	93 (39)	20	31.7	73	42.2	
Never Married, Cohabitating	22 (9)	7	11.1	15	8.7	
Now Married	84 (36)	22	34.9	62	35.8	
Divorced	27 (11)	9	14.3	18	10.4	
Separated/Widowed	8 (3)	4	6.4	4	2.3	
Prefer Not to Answer	2 (0.01)	1	1.6	1	0.6	
Education						0.0041 **
High School Graduate or Less	52 (22)	23	36.5	29	16.7	
Any College	137 (58)	31	49.2	106	61.3	
Any Graduate School	47 (20)	9	14.3	38	22.0	
Employment status						<0.0001
Employed for Wages/Self-Employed	121 (51)	27	42.9	94	54.3	
Student ‡	78 (33)	13	20.6	65	37.5	
Homemaker/Retired	22 (9)	15	23.8	7	4.1	
Unemployed	15 (6)	8	12.7	7	4.1	
Annual Income						0.0032 **†
<$25,000	82 (35)	30	47.6	52	30.1	
≥$25,000–<$50,000	36 (15)	11	17.5	25	14.4	
≥$50,000–<$75,000	27 (11)	4	6.3	23	13.3	
≥$75,000	56 (24)	9	14.3	47	27.2	
No Answer/Missing	35 (15)	8	14.3	26	15.0	

* Wilcoxon rank-sum test for continuous characteristic of age and, unless otherwise specified, Fisher’s exact test for categorical characteristics. ** Mantel–Haenszel correlation test, incorporating the ordered nature of the characteristics. † This test excludes those participants with missing income (Conclusion: those at UC centers have higher income levels than those at ED). ‡ There were 22 students who also reported being employed for wages.

**Table 2:** IPV risk factors and correlates by care setting.

	Total	Emergency Department		Urgent Care		*p*-Value *
	*n* (%)	*n*	%	*n*	%	
Social Support						
Daily contact with someone	232 (98)	61	96.8	171	98.8	0.2899
Someone to talk to about anything	227 (96)	57	90.5	170	98.3	0.0124
Someone to stay with in emergency	224 (95)	56	88.9	168	97.1	0.0178
Enough money to meet your needs	206 (87)	47	74.6	159	91.9	0.0014
Health Status						0.0028 **
Excellent	29 (12)	8	12.7	21	12.1	
Very good	83 (35)	16	25.4	67	38.7	
Good	79 (33)	13	20.6	66	38.2	
Fair	39 (17)	24	38.1	15	8.7	
Poor	6 (3)	2	3.2	4	2.3	
Limited Activities	62 (26)	29	46.0	33	19.1	<0.0001
Anxiety Diagnosis	81 (34)	31	49.2	50	28.9	0.0051
Depression Diagnosis	95 (40)	34	54.0	61	35.3	0.0110
Tobacco Use	50 (21)	17	27.4	33	19.1	0.2052
Alcohol Abuse (AUDIT-C)	135 (57)	27	42.9	108	62.4	0.0111
Drug Abuse (DAST-10)	10 (4)	4	6.6	6	3.5	0.2916

* Unless specified otherwise, Fisher’s exact test for categorical characteristics. ** Mantel–Haenszel correlation test, incorporating the ordered nature of the characteristics.

**Table 3:** Lifetime and past-year/past-partner IPV by care setting.

	Total	Emergency Department		Urgent Care		*p*-Value *
	*n* (%)	*n*	%	*n*	%	
CAS						
Physical	41 (17)	17	27.9	24	13.9	0.0184
Emotional	55 (23)	19	31.7	36	20.8	0.1117
Harassment	34 (14)	12	19.7	22	12.7	0.2066
Severe	22 (9)	10	16.7	12	7.0	0.0390
Lifetime						
Physical Abuse	68 (29)	27	43.6	41	23.7	0.0052
Threatened Physical Abuse	64 (27)	27	43.6	37	21.5	0.0014
Sexual Abuse	28 (12)	14	23.0	14	8.1	0.0047
Past Year						
Physical or Sexual Abuse	10 (4)	4	6.5	6	3.5	0.2974
Injury from Physical or Sexual Abuse	6 (3)	3	4.8	3	1.7	0.1894

* Fisher’s exact test for categorical characteristics.

**Table 4:** Frequency of IPV screening questions asked during healthcare visits.

Abuse Screening Question	Frequency of Use	Response Recorded in Medical Record
Do you feel that you are treated well by your partner/spouse/family member?	20 (8%)	All responses were “YES”
Are you or have you been threatened or abused physically, emotionally, or sexually by a partner/spouse/family member?	17 (7%)	All responses were “NO”
Has anyone ever threatened to hurt your children or your pets?	17 (7%)	All responses were “NO”
Does anyone try to keep you from having/contacting other friends or doing things outside your home?	17 (7%)	All responses were “NO”
Do you feel unsafe going back to the place where you are living?	19 (8%)	All responses were “NO”
Are you being hurt, hit, or frightened by anyone at your home or in your life?	61 (26%)	All responses were “NO”
Are there observable signs of abuse?	11 (5%)	All responses were “NO”
No abuse questions were asked	50 (21%)	N/A
Electronic medical record not located/accessed	24 (10%)	N/A

Note: No positive screens for any of the IPV questions were observed in the sample.

**Table 5:** Odds ratios of lifetime physical or sexual IPV by sociodemographic characteristics and health.

	Adjusted OR * (95% CI)	*p*-Value **
Sociodemographic characteristics		
Age, 5-year increase	0.99 (0.90, 1.10)	0.93
Race/Ethnicity		0.20
Non-Hispanic White	1.00 (Reference)	
Hispanic/Non-Hispanic Other	2.05 (0.69, 6.04)	
Marital Status		0.08
Never Married, Not Cohabitating	1.00 (Reference)	
Never Married, Cohabitating	0.76 (0.26, 2.23)	
Now Married	0.72 (0.37, 1.43)	
Divorced	2.70 (1.09, 6.66)	
Separated/Widowed	1.41 (0.28, 7.10)	
Education		0.65
High school Graduate or Less	0.79 (0.38, 1.65)	
Any College	1.00 (Reference)	
Any Graduate School	1.21 (0.59, 2.49)	
Employment status		0.80
Employed for Wages/Self-Employed	1.00 (Reference)	
Student ‡	0.85 (0.45, 1.60)	
Homemaker/Retired	0.60 (0.21, 1.73)	
Unemployed	0.81 (0.24, 2.73)	
Annual Income		0.92
<$25,000	1.00 (Reference)	
≥$25,000–<$50,000	0.99 (0.43, 2.29)	
≥$50,000–<$75,000	0.72 (0.26, 1.94)	
≥$75,000	0.89 (0.42, 1.88)	
Social Support and Health Status		
No daily contact with someone	1.00 (0.08, 12.03)	0.99
No one to talk to about anything	2.27 (0.47, 10.98)	0.31
No one to stay with in emergency	1.13 (0.30, 4.37)	0.85
Not enough money to meet your needs	1.93 (0.84, 4.44)	0.12
Health Status		0.48
Excellent, Very Good, or Good	1.00 (Reference)	
Fair or Poor	1.30 (0.63, 2.71)	
Limited Activities	2.86 (1.52, 5.39)	0.0012
Anxiety Diagnosis	3.01 (1.66, 5.44)	0.0003
Depression Diagnosis	3.99 (2.20, 7.23)	<0.0001
Tobacco Use	4.08 (2.08, 7.98)	<0.0001
Alcohol Abuse (AUDIT-C)	1.60 (0.88, 2.91)	0.12
Drug Abuse (DAST-10)	4.55 (1.07, 19.44)	0.04

* Each logistic regression adjusts for care setting (ED or UC). ** The c-statistics from the logistic regression models range from 0.61 to 0.71, with depression and tobacco use having the highest discriminatory ability. ‡ There were 22 students who also reported being employed for wages.

## Data Availability

The data presented in this study are available on request from the corresponding author.

## Appendix A

Per our a priori analysis plan, we examined whether the associations between participant characteristics and lifetime IPV varied by ED vs. UC setting. Using separate logistic regression models, a statistical interaction term between each characteristic and care setting was added to the characteristic lifetime IPV models from Table 5. Care-setting-specific odds ratios of lifetime IPV, corresponding 95% confidence intervals, and *p*-values from the likelihood ratio tests for the interaction term are presented in Table A1.

**Table A1:** Estimated odds ratios of lifetime IPV for patient characteristics by care setting.

	Emergency Department	Urgent Care	Interaction *p*-Value
	OR (95% CI)	OR (95% CI)	
Sociodemographic characteristics			
Age, 5-year increase	0.98 (0.82, 1.16)	1.00 (0.89, 1.14)	0.80
Race/Ethnicity			0.63
Non-Hispanic White	1.00 (Reference)	1.00 (Reference)	
Hispanic/Non-Hispanic Other	3.33 (0.33, 33.99)	1.74 (1.49, 6.26)	
Marital Status			0.51
Never Married, Not Cohabitating	1.00 (Reference)	1.00 (Reference)	
Never Married, Cohabitating	0.60 (0.09, 3.89)	0.96 (0.28, 3.38)	
Now Married	1.50 (0.44, 5.10)	0.51 (0.22, 1.19)	
Divorced	5.25 (0.86, 32.02)	2.12 (0.73, 6.13)	
Separated/Widowed	3.00 (0.23, 38.88)	0.88 (0.09, 9.00)	
Education			0.99
High school Graduate or Less	0.83 (0.28, 2.51)	0.76 (0.28, 2.07)	
Any College	1.00 (Reference)	1.00 (Reference)	
Any Graduate School	1.25 (0.28, 5.59)	1.19 (0.52, 2.72)	
Employment status			0.22
Employed for Wages/Self-Employed	1.00 (Reference)	1.00 (Reference)	
Student	0.50 (0.13, 1.93)	1.01 (0.48, 2.10)	
Homemaker/Retired	0.33 (0.09, 1.25)	1.24 (0.23, 6.80)	
Unemployed	0.24 (0.04, 1.45)	2.32 (0.48, 11.12)	
Annual Income			0.45
<$25,000	1.00 (Reference)	1.00 (Reference)	
≥$25,000–<$50,000	1.37 (0.34, 5.49)	0.80 (0.28, 2.28)	
≥$50,000–<$75,000	3.43 (0.32, 36.83)	0.43 (0.13, 1.47)	
≥$75,000	1.43 (0.32, 6.39)	0.71 (0.29, 1.69)	
Social Support and Health Status			
No daily contact with someone	NA	NA	
No one to talk to about anything	3.33 (0.33, 33.99)	1.48 (0.13, 16.69)	0.63
No one to stay with in emergency	1.61 (0.25, 10.40)	0.73 (0.08, 6.68)	0.59
Not enough money to meet your needs	3.38 (0.92, 12.33)	1.19 (0.35, 4.01)	0.25
Health Status			0.34
Excellent/Very Good/Good	1.00 (Reference)	1.00 (Reference)	
Fair/Poor	0.92 (0.33, 2.56)	1.84 (0.68, 5.03)	
Limited Activities	4.22 (1.44, 12.36)	2.29 (1.02, 5.11)	0.37
Anxiety Diagnosis	2.73 (0.97, 7.69)	3.15 (1.53, 6.48)	0.83
Depression Diagnosis	5.78 (1.91, 17.44)	3.40 (1.67, 6.91)	0.43
Tobacco Use	1.64 (0.53, 5.12)	6.24 (2.76, 14.10)	0.06
Alcohol Abuse (AUDIT-C)	2.08 (0.74, 5.81)	1.40 (0.68, 2.90)	0.54
Drug Abuse (DAST-10)	2.00 (0.17, 23.34)	6.35 (1.12, 35.97)	0.45

Note: Each model included main effect terms for care setting, patient characteristic, and the multiplicative interaction between care setting and patient characteristic.
